# Heat transfer analysis in a non-Newtonian hybrid nanofluid over an exponentially oscillating plate using fractional Caputo–Fabrizio derivative

**DOI:** 10.1038/s41598-022-21082-x

**Published:** 2022-11-15

**Authors:** Sami Ul Haq, Naveed Mahmood, Saeed Ullah Jan, Sehra, Ilyas Khan, Abdullah Mohamed

**Affiliations:** 1 Department of Mathematics, Islamia College Peshawar, Peshawar, Khyber Pakhtunkhwa 25000 Pakistan; 2 Department of Mathematics, Shaheed Benazir Bhutto Women University Peshawar, Peshawar, Khyber Pakhtunkhwa 25000 Pakistan; 3 Department of Mathematics, College of Science, Al-Zulfi, Majmaah University, Al-Majmaah, 11952 Saudi Arabia; 4 Research Centre, Future University in Egypt, New Cairo, 11835 Egypt

**Keywords:** Mathematics and computing, Physics

## Abstract

In this paper, we have been study a hybrid nanofluid over an exponentially oscillating vertical flat plate. Therefore the fractional derivatives definition of Caputo–Fabrizio approach is applied to transform the classical model for this hybrid nanofluid to fractional model. Together with an oscillating boundary motion, therefore the heat transfer is cause as a result of the buoyancy force produce due temperature differences between the plate and the fluid. The dimensionless classical model is generalized by transforming it to the time fractional model using Caputo–Fabrizio time fractional derivative. Exact analytical solutions are obtained by using Laplace transform method to the set of dimensionless fractional governing equations, containing the momentum and energy equations subjected to the boundary and initial conditions. Numerical computations and graphical illustrations are used to checked the results of the Caputo–Fabrizio time-fractional parameter, the second-grade parameter, the magnetic parameter and the Grashof numbers on the velocity field. An assessment for time spin-off is shown graphically of integer order versus fractional-order for these non-Newtonian hybrid nanofluid through Mathcad software. The fluid velocity increases for increasing the value of the fractional parameter, second-grade parameter and Grashof number. Also for increasing the values of the MHD parameter the fluid velocity decreases.

## Introduction

The idea of fractional-order derivatives is extremely useful in everyday life. Non-integer-order derivatives have existed from the beginning of time, just like integer order derivatives. This topic was first bounded to the field of mathematics, so the idea of fractional order derivative was widely employed in other domains a few years later. Electrochemistry, neuron models in biology, fluid mechanics, applied mathematics, shear modulus and fluid dynamics are only a few of the disciplines where this subject has been developed recently^1,2^. To examine visco-elasticity in polymers during the glassy state and in the glass transition, models were previously widely used with fractional-order derivatives^3^. Fractional derivatives were originally thought to be an effective method for obtaining a useful generalization of physical concepts. The Caputo–Fabrizio and Riemann–Liouville non-integer order derivatives are the most normally employed fractional-order derivatives. It is commonly recognized that such approaches have some applicability limitations. In the non-existence of bodily guarantee of the Riemann–Liouville derivative, the Laplace transform has terms because a constant’s of the derivative is non-zero and has a singular kernel according to Riemann–Liouville formulation. The Caputo operator solves these problems; in this approach, a power-law kernel is applied; Caputo fractional derivatives, the kernel remains singular, however, the derivative of constant is 0^4–7^.

A non-singular definition with an exponential kernel was recently proposed by Caputo–Fabrizio. To obtain an exact solution of the Caputo–Fabrizio fractional-order derivative, the Laplace transform approach can be used. Several fractional derivative models have been constructed as a result of the development of many real fluid models, which has piqued the importance of academics in the field of mathematics. The 2nd grade model of fractional fluid, Maxwell model of fractional flui, non-integer fluid model of Oldroyd, Burger fractional model of fluid and others are among the most well-known models. The heat transfer and mass transfer characteristic Jeffrey and Oldroyd-B of non-Newfluid over a stretching sheet study by^8^. Therefore Maxwell, Oldroyd and Burger are fluid models from a stem of rate type fluids, while the differential type fluids model is grade 2nd fluid is a sub-model^9,10^. Tan and Mingyu investigate a non-integer model for generalized grade 2nd fluid flow among two plates parallel^11^. The generalized 2nd grade fluid flow of natural convection is investigated using the definition of Caputo–Fabrizio by^12,13^. The non-integer model fluid between two parallel plates Maxwell and Tan et al.^14^. A viscoelastic model of fluid with regular free convection was examined using a fractional Maxwell fluid model^15^. Yin et al. investigated a viscoelastic fluid in a channel with oscillating flow using a fractional Maxwell model^16^.

The base fluids such as water, ethylene glycol, engine oil, and ethylene/water mixtures are commonly used fluids for the preparation of hybrid nanofluids. The size of the hybrid nanoparticles is very important and it should be less than 100 nm for achieving the stable hybrid nanofluids. A suspended hybrid nanoparticless over melting surface and presence of magnetic effect were study by^17,18^. Therefor the single phase of fluids such as base fluid, ethylene glycol (EG), propylene glycol (PG) and engine oil (EO) are primarily used in, among many other applications, in electronic cooling, engine cooling and vehicle thermal management, generator cooling, in machining coolant, welding, power systems, lubrication, thermal storage, solar heating, cooling and heating in buildings, biomedical, spacecraft devices and defense equipment. To achieve improved heat transfer rates with these single-phase fluids in heat exchange applications is practically impossible due to their relatively low thermal conductivities; moreover, their use with phase change is often precluded for technological or operational reasons. Therefore, one path to enhance their heat transfer performance is to disperse small quantities of nanometer-size particles^19–22^.

Researchers have observed higher heat transfer rates by using a variety of nanoparticles in different base fluids^23^. Many researchers show experimentally and computationally the heat transform properties of several hybrid nanofluids under various operation conditions. This section covers only a few of them. Hybridizing metallic nanoparticles in tiny amounts is the best cost-effective switching option for high heat transfer rates. Suresh et al.^24,25^ investigate the heat transform characteristic, conduct experiments with a $Al 2O 3-Cu$/water hybrid nanofluid. The convective heat transfer coefficient increases with the Reynolds number according to their findings. In comparison to pure water, the Nusselt number of hybrid nanofluid is increased by $13.56\%$.

Madhesh et al.^26,27^ investigated the heat transfer potential and rheological features of a CuTiO2/water hybrid nanofluid in a counter flow heat exchanger in an experimental investigation. As per their studies, the Nusselt number is the convective heat transfer coefficient, and the total heat transfer coefficient is all enhanced by 48.7%, 51.9%, and 68.0% at 1.03 volume. They carried out a comparative study on hollow cylindrical heat exchangers that use the same hybrid nanofluid and found that the thermic efficiency increased by 48.4%.

Balla et al.^28^ explored the fluid flow and heat transfer properties of a Cu-CuO/water hybrid nanofluid. As per the findings, the nano-particle nature and amount have a significant impact on the heat transfer coefficient. By raising Cu nanoparticle volume concentration in a hybrid nanofluid at a given Reynolds number, therefore a Prandtl number rises. They followed by the Nusselt number and the related convective heat transfer coefficient. When compared to water, the Nusselt number of At 1 volume fraction, the rate of heat transfer of the Cu-CuO/water hybrid nanofluid is raised by 35% and the convective heat transfer coefficient is enhanced by 40%.

The available literature is relatively scarce in what emphasises the formation of hybrid nanofluids and the finding of their thermal properties, heat transfer and friction factor. Suresh et al.^25^ prepared Al2O3-Cu hybrid nanofluids and obtained heat transfer enhancement of $13.56\%$ for 0.1% vol at a Reynolds number of 1730, while Madhesh et al.^27^, with Cu-TiO2 hybrid nanofluids, obtained heat transfer enhancement of 52% for 2.0% vol Sundar et al.^29^ prepared nanodiamond-nickel (ND-Ni) nanocomposite (hybrid) nanofluids and determined practically the thermal conductance and viscosity. Sundar et al.^29^ also prepared MWCNT-Fe3O4 hybrid nanofluids and found heat transfer enhancement of 31.10% with a pumping penalty of 18% for 0.3% vol at a Reynolds number of 22000. These studies clearly indicate that hybrid nanofluids yield higher heat transfer enhancement than single nanoparticles-based nanofluids. However, to fully understand the hybrid nanofluids mechanisms enhancing heat transfer, further experiments and analyses will be required.

In the above literature no one has considered hybrid nanofluid over exponentially oscillating using the CF time fractional derivative to find the closed form solutions. In this article, we considered the flow of hybrid nanofluid the flow is generated due to the motion along vertical plate. The purpose of current research is to investigate the heat transfer analysis of an exponentially oscillating plate of non-Newtonian hybrid nano-fluid flow over an infinite vertical flat plate using the Caputo–Fabrizio definition of fractional derivative. Magneto hydrodynamic flow is produced due to the heat transmission of the plate and the fluid. The momentum and energy equations of heat transfer have been solved using the Caputo–Fabrizio fractional derivative formulation. Therefore the Laplace transform approach was applied to solve the governing equations. Many physical parameters, such as the fractional parameter $\alpha$, the Grashof number Gr, and the magnetic parameter M are graphically investigated.

## Mathematical formulation

Consider a non-Newtonian hybrid nanofluid that is incompressible and lies over an firm flat plate of an infinite length of $xy-$plane is holding. Therefore on the $y-$axis the plate is aligned. At beginning the fluid and the plate are at rest, with a surrounding fluid temperature of $T_\infty$. Therefore the plate start to oscillate after time $t=0+$ in its own plane and with velocity $fH(t)exp(\iota \omega t)$ motion generates in the fluid , where *f* is the velocity with constant of dimension, the unit step function is *H*(*t*) and $\omega$ is the oscillation frequency. The temperature of the plate is also raised to $T_w$ (wall temperature), which is then kept at a constant level. The function *y* and *t* are assumed to be only for velocity and temperature . Additionally, a magnetic field is applied normal to the sheet and the induced magnetic field is neglected, which is justified for MHD flow at small magnetic Reynolds number. By considering the hybrid nanofluid, it is assumed that the size of nanoparticles is uniform, and the effect of the agglomeration of nanoparticles on the thermal physical properties is neglected because the nanofluid is synthesized as a stable mixture of the base fluid and nanoparticles. The constraint of deformation is satisfied exactly the same way for such a flow. The following pair of partial differential equations governs the flow behavior using Boussinesq’s approximation^30,31^.

1 $$\begin{aligned}&\rho _{hnf}\frac{\partial u(y,t)}{\partial t} =\left( \alpha _1\frac{\partial }{\partial t}+\mu _{hnf}\right) \frac{\partial ^2 u(y,t)}{\partial y^2}-\sigma _{hnf}\beta _0 u(y,t) +g(\nu \beta _T)_{hnf}( T(y,t)- T_{\infty });\quad y,t>0, \end{aligned}$$

2 $$\begin{aligned}&\frac{\partial T(y,t)}{\partial t}=\frac{\kappa }{\rho c_p}_{hnf} \frac{\partial ^2 T(y,t)}{\partial y^2};\quad y,t>0, \end{aligned}$$

Where the velocity of hybrid nano-fluid is *u*(*y*, *t*), the temperature of hybrid nanofluid is *T*(*y*, *t*), the kinematic viscosity of the hybrid nanofluid is $\nu _{hnf}$, the constant density of hybrid nanofluid is $\rho _{hnf}$, $\alpha _1$ is the non-Newtonian grade-2 parameter of fluid, *g* is the gravitational acceleration of hybrid nanofluid, $\beta _T$ is the volumetric coefficient of thermal expansion of hybrid nanofluid, $c_p$ is the heat capacity of a hybrid nanofluid at constant pressure and $\kappa$ is the hybrid nanofluid thermal conductivity. The suitable boundary and initial conditions as:

3 $$\begin{aligned}&u(y,0)=0, \quad T(y,0)=T_\infty ; \quad y>0, \end{aligned}$$

4 $$\begin{aligned}&u(0,t)=fH(t)exp(\iota \omega t), \quad T(0,t)=T_w; \quad t>0, \quad f>0, \end{aligned}$$

5 $$\begin{aligned}&u(y,t)\rightarrow 0, \quad T(y,t)\rightarrow T_\infty ; \quad y\rightarrow \infty , t>0. \end{aligned}$$

we introduce below the dimensionless variable as:

6 $$\begin{aligned} t^*&=\frac{f^2 t}{\nu }, \quad y^*=\frac{fy}{\nu },\quad u^* =\frac{u}{f}, \quad \alpha _2=\frac{\alpha _1 f^2}{\mu \nu },\nonumber \\ \theta&=\frac{T -T_\infty }{T_w -T_\infty }, \quad \text {Gr} =\frac{\nu g \beta _T(T_w-T_\infty )}{f^3}, \quad \text {Pr}=\frac{\mu C_p}{k}, \end{aligned}$$

The following dimensionless variables in (6)insert into the governing Eqs. (1) and (2) along with the boundary condition and initial conditions (3–5), we get the dimensionless problem by eliminating the star symbol.

7 $$\begin{aligned}&\frac{\partial u(y,t)}{\partial t}= A_1 \frac{\partial ^2 u(y,t)}{\partial y^2}+\alpha _2 A_2 \frac{\partial ^3 u(y,t)}{\partial t \partial y^2}+A_3 \text {Gr}\theta (y,t)-A_4 M u(y,t), \end{aligned}$$

8 $$\begin{aligned}&\frac{\partial \theta (y,t)}{\partial t}=\frac{C^*}{\text {Pr}} \frac{\partial ^2 \theta (y,t)}{\partial y^2}, \end{aligned}$$

The dimensionless boundary and initial condition are:

9 $$\begin{aligned}&u(y,0)=0, \quad \theta (y,0)=0, \quad y\ge 0, \end{aligned}$$

10 $$\begin{aligned}&u(0,t)=H(t) e^{\iota \omega t}, \quad \theta (0,t)=1, \quad t>0, \end{aligned}$$

and

11 $$\begin{aligned} u(y,t)\rightarrow 0, \quad \theta (y,t)\rightarrow 0, \quad y\rightarrow \infty ,\quad t>0. \end{aligned}$$

Here

$$\begin{aligned}&\alpha _2=\frac{\alpha _1 f^2}{\mu \nu }, \quad A_1 =\frac{\mu _{hnf}\rho }{\mu \rho _{hnf}}, \quad A_2=\frac{\rho }{\rho _{hnf}},\\&A_3=\frac{(\beta _T)_{hnf}}{\beta _T},\quad A_4 =\frac{\sigma _{hnf}\rho }{\sigma \rho _{hnf}}, \quad \text {M}=\frac{\sigma \nu {\beta _0}^2}{\rho f^2},\\&\text {C}^*=\frac{k_{hnf}\rho c_p}{k(\rho c_p)_{hnf}}. \end{aligned}$$

M is magnetic parameter , $\alpha _2$ is the 2nd grade parameter, Gr is the Grashof number and Pr is the Prandtl number.

In ways to construct a model with a CF time fractional derivative define in (14). We use this Caputo–Fabrizio fractional derivative instead of time dependent derivative therefore replace $\frac{\partial }{\partial t}(.)$ by ${D_t}^\alpha$ in Eq. (7) and Eq. (8) we have

12 $$\begin{aligned}&{D_t}^\alpha u(y,t)=A_1 \frac{\partial ^2 u(y,t)}{\partial y^2} +\alpha _2 A_2 D^\alpha _t \alpha _2 \frac{\partial ^2 u(y,t)}{\partial y^2} +A_3 Gr \theta (y,t)-A_4 M u(y,t), \end{aligned}$$

13 $$\begin{aligned}&{D_t}^\alpha \theta (y,t)=\frac{C^*}{Pr} \frac{\partial ^2 \theta (y,t)}{\partial y^2}, \end{aligned}$$

Therefore the definition of CF time fractional derivative and the literature as

14 $$\begin{aligned} {D_t}^\alpha f(t)&= \frac{1}{1-\alpha }\int _{0}^{1}f^{'}(t). exp\left( \frac{-\alpha (t-T)}{1-\alpha }\right) dT, \quad 0<\alpha < 1, \nonumber \\ L\left[ {D_t}^\alpha f(t)\right]&= \frac{s}{s(1-\alpha )+\alpha }. \end{aligned}$$

## Analytical solution by Laplace transform

Now applying the Laplace transform to the (initial-boundary) conditions from (9–11) equations (12–13). Because the velocity equation is based on the temperature equations, we will first discover solutions for the temperature equations, and then we will apply the Laplace transform method to find solutions for the equation of velocity .

### Solution for temperature equation

Laplace transform applying the to Eq. (13) and also to the boundary condition (10)2 and (11)2, we get

$$\begin{aligned}&\frac{s}{s(1-\alpha )+\alpha }\theta (y,s)=\frac{1}{C^*Pr} \frac{\partial ^2 \theta (y,s)}{\partial y^2}\\&\theta (0,s)=\frac{1}{s},\quad \theta (y,s)\rightarrow 0, \quad \text {as}\quad y\rightarrow \infty \end{aligned}$$

use the symbols $\gamma =\frac{1}{1-\alpha }$, we have

15 $$\begin{aligned}&\frac{C^*Pr\gamma s}{s+\alpha \gamma }\bar{\theta }(y,s) =\frac{\partial ^2\bar{\theta }(y,s)}{\partial y^2}, \end{aligned}$$

16 $$\begin{aligned}&\theta (0,s)=\frac{1}{s},\quad \theta (y,s)\rightarrow 0 \quad \text {as}\quad y\rightarrow \infty , \end{aligned}$$

Solution of the transform Eqs. (15) and (16) is

17 $$\begin{aligned} \bar{\theta }(y.s)=\frac{1}{s}exp \left( -y\sqrt{\frac{C^* Pr \gamma s}{s+\alpha \gamma }}\right) =\Phi (y,s;C^* Pr\gamma ,\alpha \gamma ) \end{aligned}$$

Respectively, Hence we define the temperature field as follows:

18 $$\begin{aligned} \theta (y,t)=\varphi (y,t;C^*Pr\gamma ,\alpha \gamma ),\quad 0<\alpha <1 \end{aligned}$$

Hence $\Phi (y,s;C^*Pr\gamma ,\alpha \gamma )$ and $\varphi (y,t;C^*Pr\gamma ,\alpha \gamma )$ are define an the appendices.

### Solution for dimensional velocity equation

Now the Laplace Transform(LT) method applying to the dimensional Eq. (12), as well as the initial condition and also to the boundary conditions (9–11), we get

19 $$\begin{aligned} \left( \frac{\gamma s+A_4 M(s+\alpha \gamma )}{s+\alpha \gamma }\right) \bar{u}(y,s)&= \left( \frac{A_1(s+\alpha \gamma )+\alpha _2 A_2 \gamma s}{s+\alpha \gamma }\right) \frac{\partial ^2 \bar{u}(y,s)}{\partial y^2} \nonumber \\&\quad +A_3 Gr\frac{1}{s} exp\left( -y\sqrt{\frac{c^* Pr \gamma s}{s+\alpha \gamma }}\right) \end{aligned}$$

20 $$\begin{aligned} \bar{u}(y,s)&=\frac{1}{s-\iota \omega },\quad \bar{u}(y,s)\rightarrow 0 \quad \text {as}\quad y\rightarrow \infty . \end{aligned}$$

Equation (19) represented solution is the partial differential equation’s , and the boundary condition (20) does have the solution.

21 $$\begin{aligned} \bar{u}(y,s)&=\frac{1}{s-\iota \omega }exp \left( -y\sqrt{\frac{(\gamma +A_4M)s+A_4M\alpha \gamma }{(A_1+\alpha _2 A_2 \gamma )s +A_1\alpha \gamma }}\right) +k_5\frac{1}{s}exp\left( -y\sqrt{\frac{(\gamma +A_4M)s +A_4M\alpha \gamma }{(A_1+\alpha _2 A_2 \gamma )s+A_1\alpha \gamma }}\right) \nonumber \\&\quad +k_6\frac{1}{s+k_3}exp\left( -y\sqrt{\frac{(\gamma +A_4M)s+A_4M\alpha \gamma }{(A_1+\alpha _2 A_2 \gamma )s+A_1\alpha \gamma }}\right) +k_7\frac{1}{s+k_4}exp \left( -y\sqrt{\frac{(\gamma +A_4M)s+A_4M\alpha \gamma }{(A_1+\alpha _2 A_2 \gamma )s +A_1\alpha \gamma }}\right) \nonumber \\&\quad -k_5\frac{1}{s}exp\left( -y\sqrt{\frac{C^* \text {Pr} \gamma s }{s+\alpha \gamma }} \right) -k_6\frac{1}{s+k_3} exp\left( -y\sqrt{\frac{C^* \text {Pr} \gamma s }{s+\alpha \gamma }} \right) -k_7\frac{1}{s+k_4}exp \left( -y\sqrt{\frac{C^* \text {Pr}\gamma s }{s+\alpha \gamma }}\right) \end{aligned}$$

where

$$\begin{aligned}&\alpha _2=\frac{\alpha _1 f^2}{\mu \nu }, \quad A_1 =\frac{\mu _{hnf}\rho }{\mu \rho _{hnf}}, \quad A_2=\frac{\rho }{\rho _{hnf}},A_3=\frac{(\beta _T)_{hnf}}{\beta _T},\\&A_4=\frac{\sigma _{hnf}\rho }{\sigma \rho _{hnf}},\quad \text {M} =\frac{\sigma \nu {\beta _0}^2}{\rho f^2},\quad \text {C}^* =\frac{k_{hnf}\rho c_p}{k(\rho c_p)_{hnf}},\\&k_3=\frac{1}{2}\left( k_1-\sqrt{k^2_1-4k_2}\right) , \quad k_4=\frac{1}{2}\left( k_1+\sqrt{k^2_1-4k_2}\right) \\&k_1=\frac{\alpha \gamma ^2(c^*A_1Pr-1)-2A_4 M\alpha \gamma }{c^*Pr\gamma (A_1+A_2\alpha \gamma )-(\gamma +A_4 M)},\quad k_2=\frac{-A_4M\alpha ^2\gamma ^2}{c^*Pr\gamma (A_1+A_2\alpha \gamma )-(\gamma +A_4 M)}\\&k_5=\frac{A_3Gr\alpha ^2\gamma ^2}{k_3 k_4(c^* Pr\gamma (A_1+A_2\alpha \gamma ) -(\gamma -A_4M))},\\&k_6=\frac{A_3Gr(k^2_3-2\alpha \gamma k_3+\alpha ^2 \gamma ^2)}{k_3(k_3-k_4) (c^* Pr\gamma (A_1+A_2\alpha \gamma )-(\gamma -A_4M))},\\&k_7=\frac{A_3Gr(k^2_4-2\alpha \gamma k_4+\alpha ^2 \gamma ^2)}{k_4 (k_4-k_3)(c^* Pr\gamma (A_1+A_2\alpha \gamma )-(\gamma -A_4M))}. \end{aligned}$$

The inverse transform of the preceding velocity Eq. (21) now to find the close form solution shown below

$$\begin{aligned} u(y,t)= & {} \int _{0}^{t}(exp({\iota \omega (t-\tau )})+k_5 +k_6 e^{-k_3(t-\tau )}+k_7e^{-k_4(t-\tau )})\times h(\tau )d\tau \\&-m\varphi (y,t;c^*\text {Pr}\gamma ,\alpha \gamma )-k_6\psi (y,t;c^* \text {Pr}\gamma ,\alpha \gamma ,-k_3)\\&-k_7\psi (y,t;c^*\text {Pr}\gamma ,\alpha \gamma ,-k_4) \end{aligned}$$

here $m=k_5+k_6+k_7, \varphi (y,t;c^*Pr\gamma ,\alpha \gamma )$ and $\psi (y,t;c^*Pr\gamma ,\alpha \gamma ,-k_3)$ are define by the (1)–(6).

#### Special cases

In the absence of nano-particle and because of the simplicity of the solution one can easily compare from our solution the temperature profile is same is the Eq. (18), where is the solution for momentum equation as concerned then in the absence of nano-particle c and magnetic field our solution is similar to the solution achieving by Shah and Khan^32^.

#### Limiting cases

 In the absence of $M = 0$, we recover the result of Shah and Khan^32^.

## Graphical results and discussions

**Figure 1:**
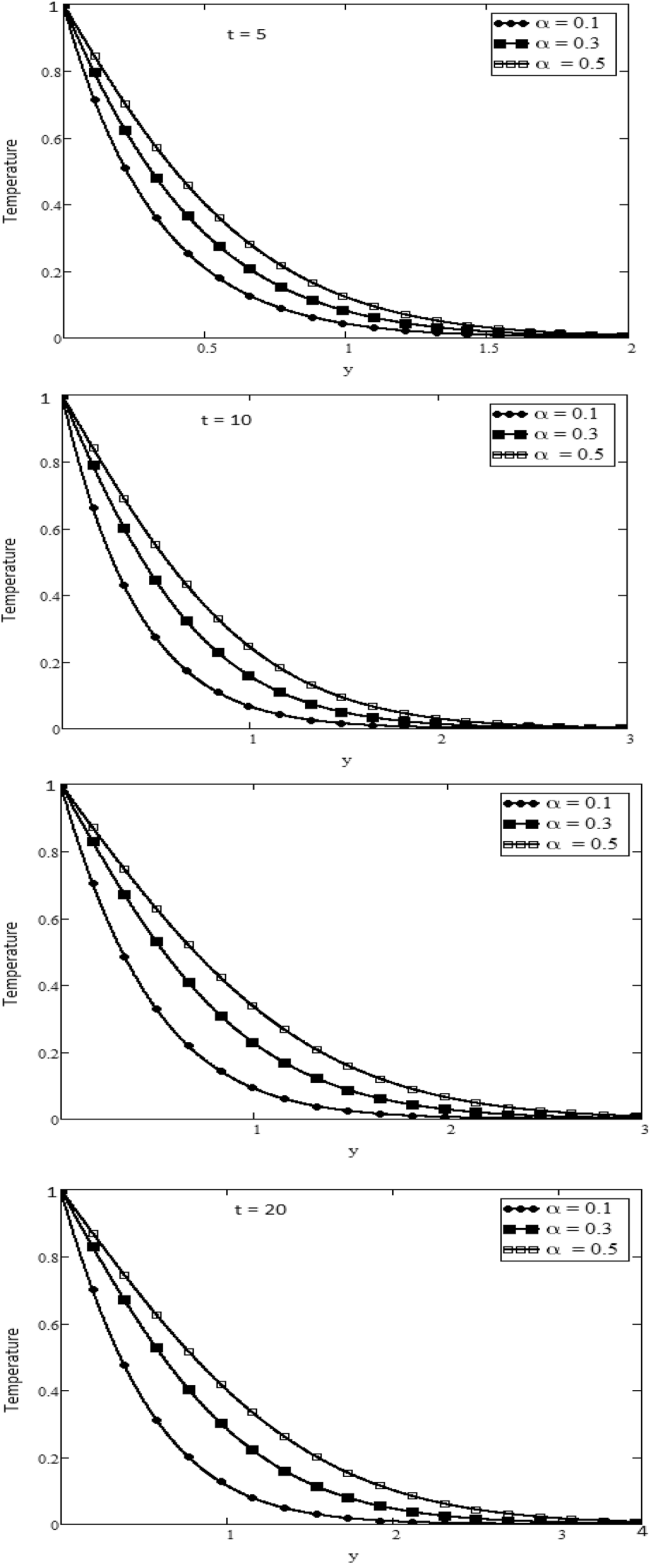
Temperature profile.

Over an infinite flat plate, a non-Newtonian hybrid nanofluid with an exponentially oscillating plate has been investigated. The dimensionless momentum and energy equations are solved using the definition of CF non-integer order derivative, and the close form solution is achieved by applying the inverse Laplace transform technique. Under the assumptions for various physical parameters for example $\alpha$, Pr, Gr, that for a physical description of the problem, the velocity and temperature curves are visibly displayed. Figure 1 Variations in temperature profiles for several fractional parameter $\alpha$ and time *t* values are shown. Figure 1 shows that the temperature has been rising as the $\alpha$ has been increased. With t and $\alpha$, the size of the thermal boundary layer grows.

**Figure 2:**
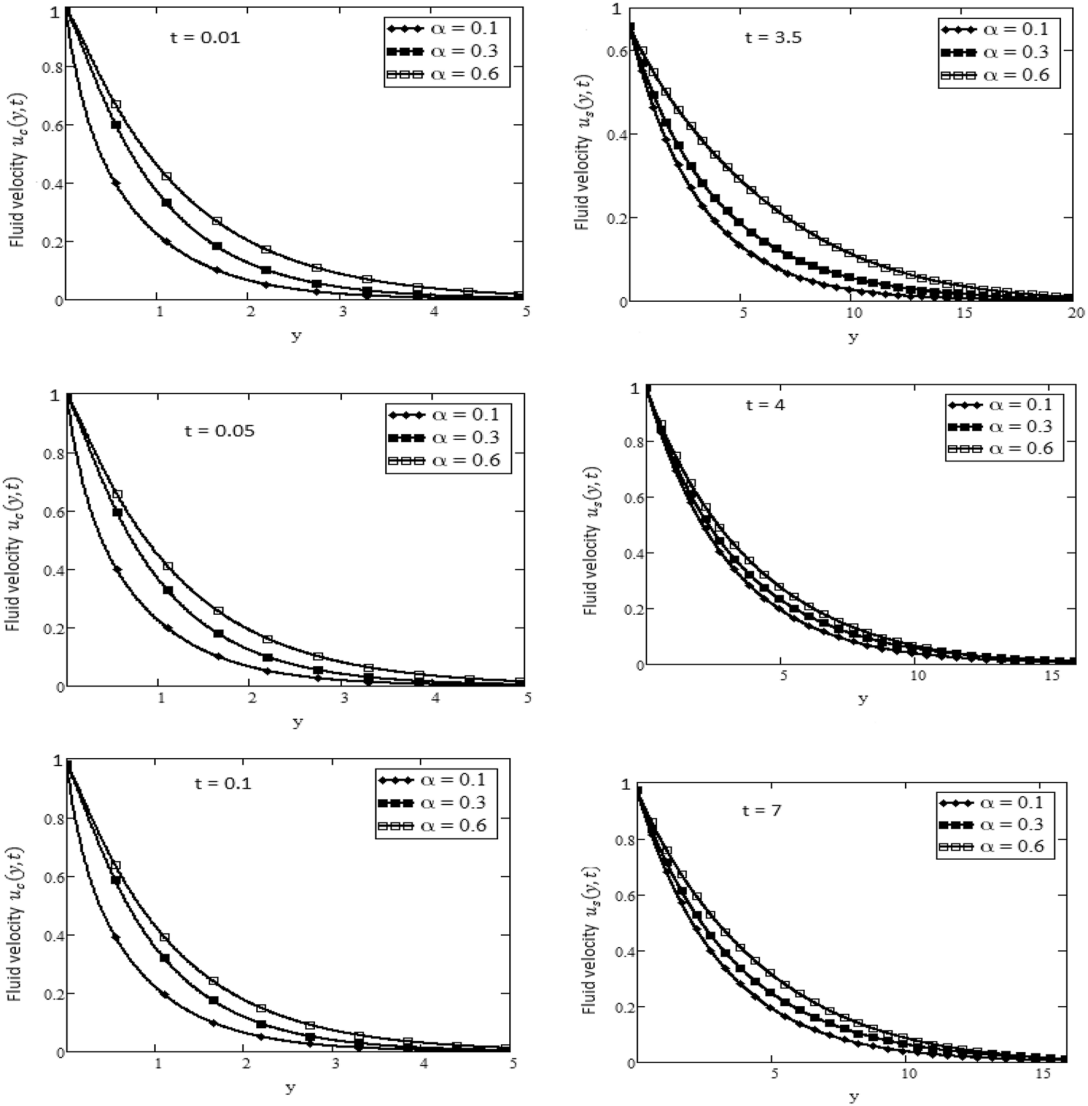
Velocity plot in the form of cosine and sine oscillation or various values of $\alpha$ of at *t*, when $\hbox {Pr} = 7$, $M = 15$.

**Figure 3:**
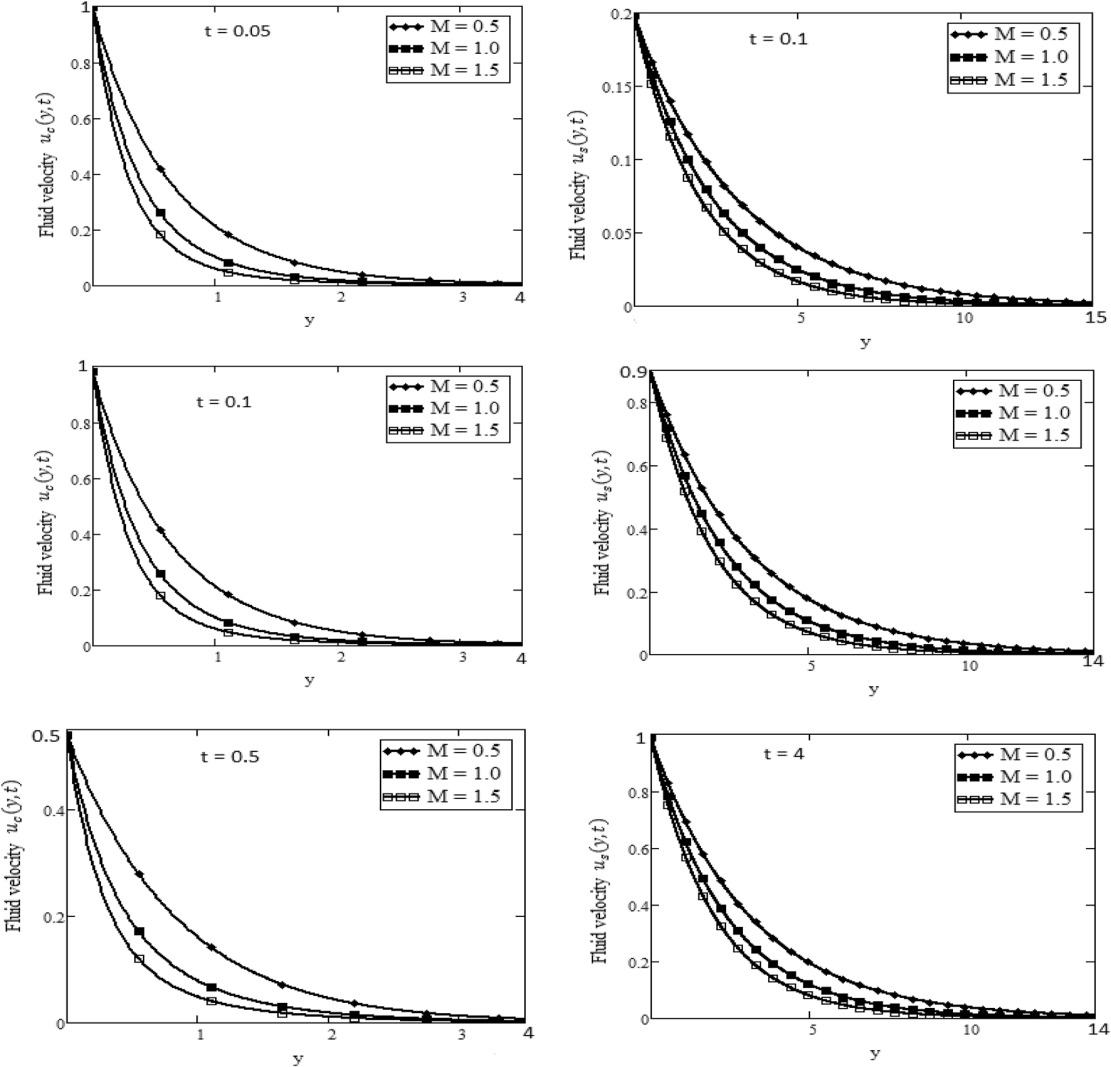
Velocity plot in the form of cosine and sine oscillation or various values of *M* of at *t*, when $\hbox {Pr} = 7$, $\alpha = 0.5$.

**Figure 4:**
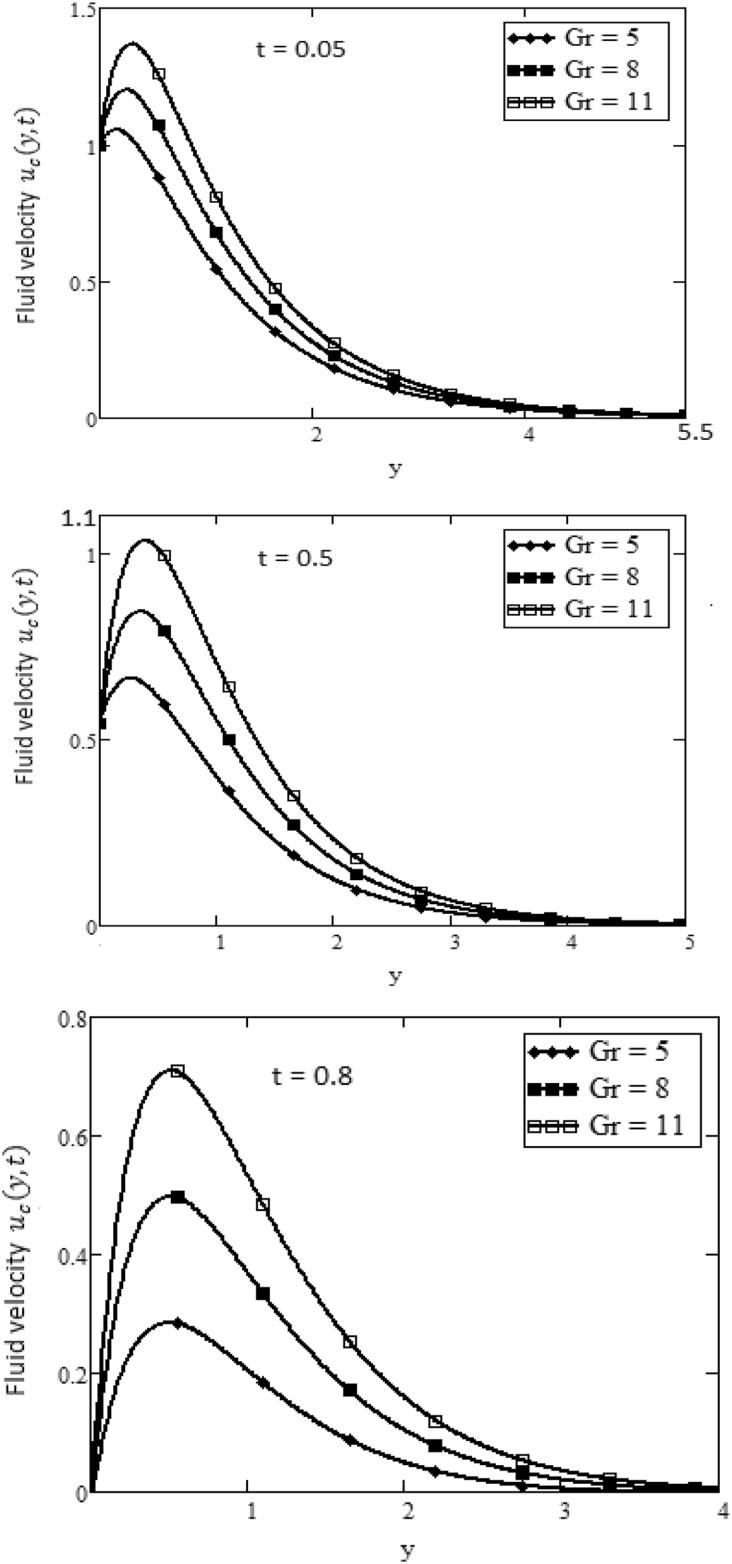
Velocity plot in the form of cosine and sine oscillation or various values of *Gr* of at *t*, when $\hbox {Pr} = 7$, $\alpha = 0.5$, $M = 15$, $\alpha _2= 2$.

**Figure 5:**
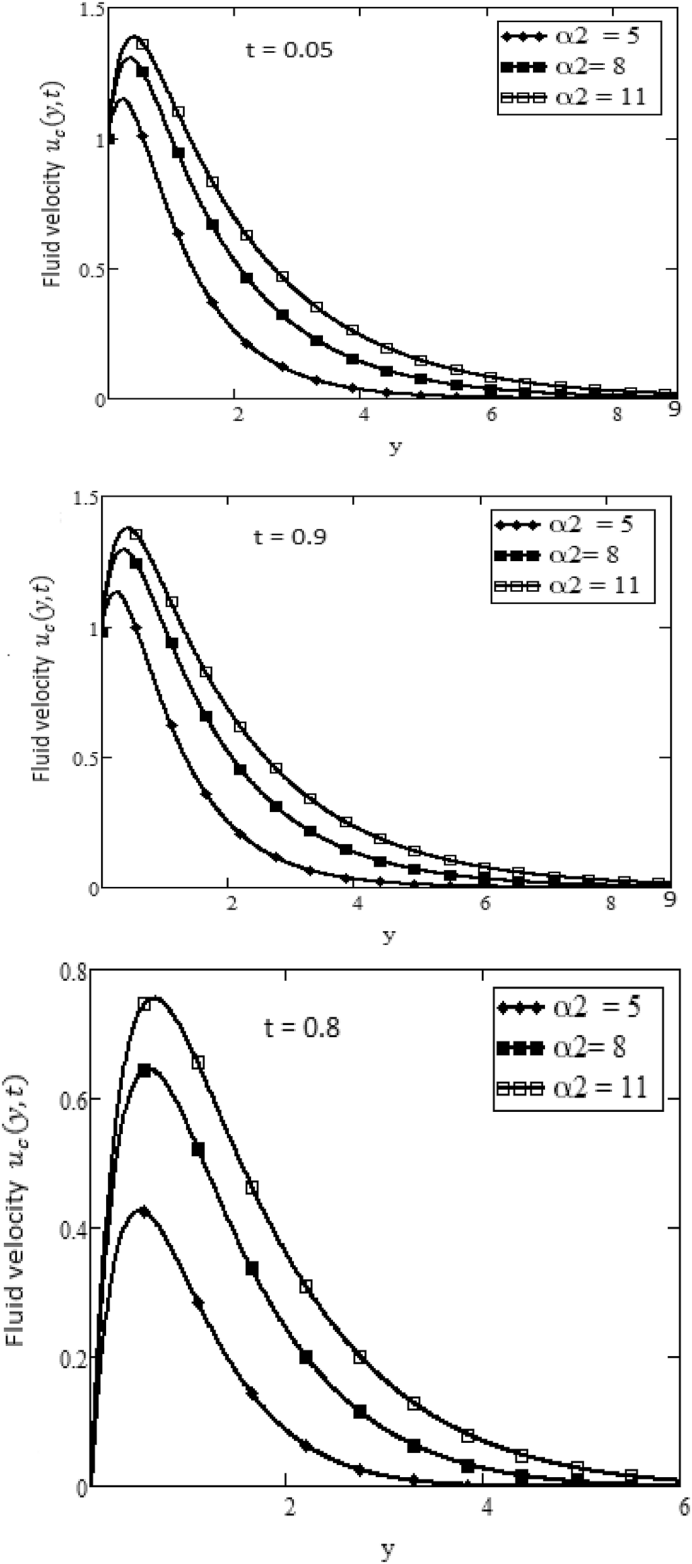
Velocity plot in the form of cosine and sine oscillation or various values of $\alpha _2$ of at *t*, when $\hbox {Pr} = 7$, $\alpha = 0.5$, $M = 15$, $Gr = 7$.

Figures 2 and 3 depict the velocity profile caused by changes in the fractional parameter $\alpha$. The plate oscillation in both cases sine and cosine oscillation was considered. Both cosine and sine oscillations were depicted in Figs. 2 and 3. Figure 2 shows that the movement of fluids raises for high-value fractional parameter $\alpha$ and time *t* and in both cases of cosine and sine oscillation. Figure 3 illustrates that when the value of time *t* and M increase, in both cases of cosine and sine oscillation the velocity fluid falls. Fluid motion rises with large values of *Gr* and larger values of *t* related to the cosine oscillations case, as shown in Fig. 4. Figure 5 demonstrates that fluid velocity rises for large values of the second-grade parameter $\alpha _2$ and large as the value of *t* in the case of cosine oscillation.

## Conclusions

This paper deal with hybrid nanofluid over an exponentially oscillating plate using Caputo–Fabrizio fractional derivative to transform the classical model to fractional dimensionless model. The Laplace transform approach was used to obtain a closed-form solution of the problem. The limiting solutions for ordinary hybrid nanofluids and non-Newtonian fluids were extracted. The findings were graphically assessed for time fractional $\alpha$, second grade parameters $\alpha _2$, magnetic parameter M, and Gr for different times. Therefore the graphical solution show that the value of the fractional parameter $\alpha$ enhance, the fluid temperature rises. Also the fluid motion raise for high values of $\alpha$ (fractional parameter) and the time *t* in both cases of a sine and cosine oscillation as well as the motion of fluid is accelerating for large value of Gr, while decelerating for large value of *t* in case of cosine oscillation.

## Data Availability

The datasets used and/or analyzed during the current study available from the corresponding author on reasonable request.

## Appendix

22 $$\begin{aligned} \phi (y,s;p,q)&= \frac{1}{s}exp\bigg (-y\sqrt{\frac{p}{s+q}}\bigg ), \nonumber \\ \phi (y,t;p,q)&= \mathcal {L}^{-1}[\phi (y,s;p,q)]=\mathcal {L}^{-1} \bigg [\frac{1}{s}exp\bigg (-y\sqrt{\frac{p.s}{s+q}}\bigg )\bigg ]\nonumber \\&=1-\frac{2p}{\pi }\int _{0}^{\infty }\frac{sin(yx)}{x(p+x^2)} exp\bigg ({-\frac{qtx^2}{p+x^2}}\bigg )dx, \end{aligned}$$

23 $$\begin{aligned} G(y,s;p,q,r)&= \frac{1}{s-b}e^{-y\sqrt{\frac{p.s}{s+q}}}\nonumber \\&=\phi (y,s;p,q)+\psi (y,s;p,q,r), \end{aligned}$$

24 $$\begin{aligned} \psi (y,s;p,q,r)&=\frac{1}{s-b}\phi (y,s;p,q), \end{aligned}$$

25 $$\begin{aligned} \psi (y,t;p,q,r)&= \mathcal {L}^{-1}[\psi (y,s;p,q,r)]\nonumber \\&=exp\bigg (kt-y\sqrt{\frac{p.r}{q+r}}-1-\frac{2p.r}{\pi } \int _{0}^{\infty }\frac{sin(yx)}{x(p.r+(q+r)x^2)} e^{-\frac{qtx^2}{p+x}}dx\bigg ), \end{aligned}$$

26 $$\begin{aligned} g(y,t;p,q,r)&=\mathcal {L}^{-1}[G(y,s;p,q,r)]=\phi (y,t;p,q,r)+\psi (y,t;p,q,r), \end{aligned}$$

27 $$\begin{aligned} h(\tau )&= \mathcal {L}^{-1}\bigg [exp\bigg (-y\sqrt{\frac{a_2.s+b_1}{s+b_2}}\bigg )\bigg ] =\delta (t)exp\big ({-y\sqrt{a_2}}\big ) +\int _{0}^{\infty }\frac{y}{2u\sqrt{\pi }}\sqrt{\frac{a_2b_2-b_1}{t}} \nonumber \\&\quad \times exp\bigg ({-\frac{y^2}{4u}}\bigg )\times exp \big ({-b_2t-a_2.u}\big ) \times I_1\bigg (2\sqrt{(a_2.b_2-b_1)ut}\bigg )du \end{aligned}$$

## List of symbols

*u*—$\left[ LT^{-1}\right] \;(\mathrm{ms}^{-1})$: Velocity of fluid
*T*—$[\theta ]\;(\mathrm{K})$: Temperature of fluid
*t*—$$[T]\;(\mathrm{s})$$: Time
$C_p$—$\left[ L^2MT^{-1}\theta ^{-1}\right] \;(\mathrm{jkg}^{-1}\,\mathrm{K}^{-1})$: Specific heat at a constant pressure
*g*—$\left[ LT^{-2}\right] \;(\mathrm{ms}^{-2})$: Acceleration due to gravity
*k*—$\left[ MLT^{-3}\theta ^{-1}\right] \;(\mathrm{Wm}^{-2}\,\mathrm{K}^{-1})$: Thermic conductivity of fluid
$\mu$—$\left[ ML^{-1}T^{-1}\right] \;(\mathrm{mkg}^{-1}\,\mathrm{s}^{-1})$: Dynamic viscosity of fluid
$\nu$—$\left[ L^2T^{-1}\right] \;(\mathrm{m}^2\,\mathrm{s}^{-1})$: Viscosity (kinematic) of the fluid
$$\rho$$—$$\left[ ML^{-3}\right] \;(\mathrm{kgm}^{-3})$$: Fluid density
$\beta _T$—$\left[ \theta ^{-1}\right] \; (\mathrm{K}^{-1})$: Thermal coefficient of volumetric expansion of fluid
$T_w$: Fluid temperature at the plat
$T_\infty$: Ambient fluid temperature
Gr: Grashof number
$$\alpha _2$$: Grade 2nd parameter
$$\alpha$$: Fractional parameter
Pr: $$\left( \frac{\mu C_p}{k}\right)$$
s: Parameter for laplace transform
